# DIVERGesTOOL – Entwicklung einer Toolbox zur Erfassung geschlechtlicher Vielfalt in der quantitativen Gesundheitsforschung

**DOI:** 10.1007/s00103-024-03915-4

**Published:** 2024-06-28

**Authors:** Sophie Horstmann, Corinna Schmechel, Eva Becher, Sabine Oertelt-Prigione, Kerstin Palm, Gabriele Bolte

**Affiliations:** 1 Institut für Public Health und Pflegeforschung, Abteilung Sozialepidemiologie, Universität Bremen, Bremen, Deutschland; 2 Institut für Geschichtswissenschaften, Humboldt-Universität zu Berlin, Berlin, Deutschland; 3 Medizinische Fakultät OWL, AG 10 Geschlechtersensible Medizin, Universität Bielefeld, Bielefeld, Deutschland; 4 Department of Primary and Community Care, Radboud Universität Nijmegen, Nijmegen, Niederlande

**Keywords:** Geschlechtersensibilität, Multidimensionalität, Datenerfassung, Fragebogen, Diversität, Sex/gender sensitivity, Multidimensionality, Data collection, Questionnaire, Diversity

## Abstract

In der epidemiologischen Gesundheitsforschung besteht ein großer Bedarf an umfassenden Erhebungsinstrumenten, die der Multidimensionalität und Variabilität von Geschlecht gerecht werden. Das Forschungsprojekt DIVERGesTOOL griff diesen in den letzten Jahren immer deutlicher werdenden Bedarf auf. Es verfolgte das Ziel, eine anwendungsorientierte Toolbox zur Erfassung geschlechtlicher Vielfalt für die quantitative Gesundheitsforschung in Deutschland zu entwickeln.

Der Entwicklungsprozess war partizipativ angelegt, Vertreter*innen großer epidemiologischer Studien in Deutschland wurden direkt einbezogen. Im Rahmen von vier gemeinsamen Workshops wurde eine Toolbox entwickelt, die sich aus mehreren Bestandteilen zusammensetzt: Die Basis-Items sind ein grundlegendes, allgemein nutzbares Set aus drei Fragen, die sich am Two-Step-Approach orientieren. Sie werden anstelle der bisher routinemäßig in den Gesundheitswissenschaften angewendeten binären Geschlechtsvariable empfohlen. Zudem enthält die Toolbox Zusatz-Items mit beispielhaften Fragebogen-Items für spezifische Fragestellungen oder Studienpopulationen. Ergänzt wurden die Items um ausführliche Anwendungshinweise und Hintergrundinformationen. Die Toolbox steht Interessierten online kostenlos über die Website des Projektes zur Verfügung (https://www.uni-bremen.de/divergestool-projekt/divergestool-toolbox).

Langfristig soll die DIVERGesTOOL-Toolbox Forschende dabei unterstützen, geschlechtliche Vielfalt in die eigene Forschung zu integrieren, und somit zu mehr Geschlechtersensibilität in der Gesundheitsforschung und validen Forschungsergebnissen beitragen.

## Abstract (en)

In health research, there is a need for comprehensive survey instruments capable of assessing the multidimensionality and variability of sex/gender. The research project DIVERGesTOOL was conducted in response to this need, which has become increasingly evident in recent years. The aim was to develop an application-oriented toolbox for the assessment of sex/gender diversity in quantitative health-related research in Germany.

The development process followed a participatory design, as representatives of large epidemiological studies in Germany were directly involved. During four collaborative workshops, a toolbox was developed that contains several different elements. The basic items are a generally usable set of three different questionnaire items based on the two-step approach. They are recommended as a replacement for the binary sex or gender item that are currently routinely used in health-related research. In addition, the toolbox contains further exemplary questionnaire items for specific research questions or study populations. The developed items were supplemented with detailed instructions for their application and additional information. The toolbox is an open online resource accessible to any user (https://www.uni-bremen.de/divergestool-projekt/divergestool-toolbox).

In the long term, the DIVERGesTOOL is intended to support researchers in integrating sex/gender diversity into their own research and thus to contribute to more sex/gender sensitivity in health-related research and valid findings.

## Hintergrund

In unserer Gesellschaft stellt das Geschlecht eine der Kategorien dar, die unser Zusammenleben erheblich prägen. Es beeinflusst die soziale Rolle einer Person, ihre Beziehungen und welche Eigenschaften und Verhaltensweisen ihr von anderen zugeschrieben und von ihr erwartet werden [1]. Darüber hinaus wirken sich gesellschaftliche Geschlechterverhältnisse deutlich darauf aus, zu welchen Ressourcen eine Person Zugang hat und welche Chancen ihr im Leben geboten werden [2]. Geschlecht hat eine große Bedeutung für die Gesundheit: So konnte in allen Bereichen von Medizin und Public Health die Wirkung der verschiedenen biologischen und sozialen Dimensionen von Geschlecht auf die Gesundheit bereits vielfältig nachgewiesen werden [2–4].

Daher rückt in der Gesundheitsforschung zunehmend die Notwendigkeit, das Geschlecht der Untersuchten systematisch zu berücksichtigen, in das Bewusstsein von Forschenden, Entscheidungstragenden und Politik. In Richtlinien für Forschungsförderungen auf nationaler und internationaler Ebene sowie für Publikationen in wissenschaftlichen Zeitschriften wird immer häufiger die angemessene Berücksichtigung von Geschlecht im gesamten Studienverlauf – von der Studienkonzeption bis zur Ergebnispräsentation und -diskussion – gefordert (siehe beispielsweise die Stellungnahme des Senats der Deutschen Forschungsgesellschaft (DFG; [5]) oder die Regelungen zu Gender und Chancengleichheit im EU-Forschungsrahmenprogramm Horizont 2020 [6]). Zahlreiche innerhalb der letzten Jahre veröffentlichten Leitlinien unterstützen Forschende mit Hinweisen für eine strukturierte und methodische Herangehensweise [7–9]. Allerdings stellt hierbei insbesondere die Operationalisierung eines multidimensionalen Konzeptes von Geschlecht nach wie vor eine Herausforderung dar.

Gemäß dem aktuellen, gendertheoretisch und gesundheitswissenschaftlich fundierten Verständnis existieren verschiedene soziale und biologische Dimensionen von Geschlecht, die miteinander in Wechselwirkung stehen, ohne sich gegenseitig zu bedingen. Die biologischen Dimensionen beziehen sich auf die körperlichen Merkmale, die mit der Reproduktion in Verbindung gebracht werden [10]. Hierzu zählen die Chromosomen, Hormone und die Anatomie einer Person in Bezug auf die äußeren und inneren Geschlechtsorgane [11, 12]. Die sozialen Dimensionen umfassen Identitäten, Beziehungen und Normen [2, 11, 12]. Sie werden auf drei Ebenen untersucht, die geschlechterbezogene Identitätskonstruktionen, Gesellschaftsstrukturen und symbolische Repräsentationen voneinander unterschieden aufschlüsseln [13]. Sowohl die biologischen als auch die sozialen Dimensionen von Geschlecht zeichnen sich durch eine große Variationsbreite aus [11, 12]. In der Praxis lassen sich die verschiedenen Dimensionen von Geschlecht nicht immer eindeutig voneinander trennen und sollten in der Forschung zusammengedacht werden [10].

Obschon das Bewusstsein für die Multidimensionalität und Variabilität von Geschlecht in der Gesundheitsforschung zunimmt, wird Geschlecht vor allem in der quantitativen, epidemiologischen Gesundheitsforschung bislang weitgehend routinemäßig über ein einziges, binäres Item erfasst, das ausschließlich zwischen den beiden distinkten Kategorien „Frau“ und „Mann“ unterscheidet [11, 14, 15]. Seit der Anpassung des Personenstandsgesetzes (PStG) im Dezember 2018 findet sich zudem vermehrt eine Ergänzung um die dritte Kategorie „divers“ und die Möglichkeit keinen Eintrag zu machen. Problematisch ist, dass diesem Ansatz einer statischen Kategorie von Geschlecht die Annahme zugrunde liegt, dass sich die Personen innerhalb einer der beiden (bzw. durch die Anpassung des PStG vier) Gruppen auch in Bezug auf andere Eigenschaften, wie der Hormonkonzentration, bestimmter Verhaltensweisen, Ansichten oder Lebensumstände, gleichen und eindeutig von den Personen der anderen Geschlechterkategorie(n) abgrenzen lassen [16, 17].

Infolge dieser vereinfachenden Kategorisierung von Geschlecht wird der Beitrag, den die einzelnen Geschlechterdimensionen für das Auftreten der zu untersuchenden gesundheitlichen Phänomene leisten, verschleiert [15, 17]. Mechanismen, die den beobachteten Einflüssen des Geschlechts auf die Gesundheit zugrunde liegen, bleiben im Verborgenen und potenzielle Wechselwirkungen zwischen den einzelnen Dimensionen von Geschlecht können durch die ausschließliche Berücksichtigung einer einzelnen Geschlechtsvariable nicht identifiziert werden [14, 18].

Innerhalb der routinemäßig verwendeten Geschlechtsabfrage wird normalerweise nicht spezifiziert, auf welche Dimension von Geschlecht sich diese Frage bezieht. Für Personen, deren Geburtsgeschlecht nicht mit ihrer geschlechtlichen Identität übereinstimmt, ergibt sich daher die Problematik, dies bei der Beantwortung des Fragebogens selbst entscheiden zu müssen. Die Konsequenz ist eine große Heterogenität der geschlechtlichen Realitäten innerhalb der einzelnen Geschlechterkategorien [19]. Somit ist es möglich, dass die folgenden Personen auf die Frage nach ihrem Geschlecht mit „Frau“ antworten:

- Personen, deren Geburtsgeschlecht und geschlechtliche Identität „Frau“ ist,
- Personen, die bei ihrer Geburt als „Frau“ eingeordnet wurden, sich aber selbst nicht als solche identifizieren,
- Personen, die sich selbst als Frau beschreiben, aber nicht als solche in ihrer Geburtsurkunde eingeordnet wurden.

Das Resultat ist eine weite Spanne sozialer und biologischer Eigenschaften, die unter der Kategorie „Frau“ undifferenziert erhoben werden, die Forschende bei dem Versuch, die mithilfe der Geschlechtsvariable gewonnenen Ergebnisse zu interpretieren, vor große Schwierigkeiten stellt [20].

Hinzu kommt, dass ein binäres Item zur Erfassung von Geschlecht, das ausschließlich die Möglichkeiten „Mann“ oder „Frau“ als Antwort anbietet, der großen geschlechtlichen Variabilität nicht gerecht wird. Es ignoriert die Existenz von Personen, die sich außerhalb oder zwischen diesen Kategorien verorten [17], und zwingt diese dazu, sich einer Kategorie zuzuordnen, die nicht auf sie zutrifft [15], oder die Frage nicht zu beantworten. Neben einer Gefährdung der Datenqualität führt eine binäre Geschlechtsvariable somit auch zum Ausschluss und zur Diskriminierung von Personen, die sich außerhalb der geschlechtlichen Binarität bewegen [21].

Es zeigt sich somit, dass ein einziges Item nicht ausreicht, um die große Komplexität und Dynamik von Geschlecht angemessen abzubilden, insbesondere wenn dieses Item ausschließlich die zwei distinkten Antwortoptionen „Mann“ und „Frau“ berücksichtigt. Eine unzureichende Abbildung von Geschlecht kann zu verzerrten Forschungsergebnissen führen [21]. Es ist längst überfällig, dass die unterschiedlichen Dimensionen von Geschlecht durch den Einsatz komplexer, differenzierter, theoriebasierter und zugleich handhabbarer Erhebungsinstrumente konsequenter in die Forschung integriert werden [17, 22].

In Deutschland finden sich erste Ansätze, um Geschlecht differenzierter in für die Gesundheitsforschung relevanten Befragungen auf Bevölkerungsebene zu erfassen. So wurde im Jahr 2019 vom Robert Koch-Institut erstmals in der Gesundheitsbefragung „Gesundheit in Deutschland aktuell“ (GEDA 2019/2020-EHIS) eine zweistufige Geschlechtsabfrage verwendet, die sowohl den Geschlechtseintrag in der Geburtsurkunde als auch die geschlechtliche Identität berücksichtigt [23]. Im Rahmen des Forschungsprojektes INGER wurden verschiedene Items zur Abfrage geschlechtlicher Dimensionen in die im Rahmen der KORA-Studie im Jahr 2019 durchgeführten Befragungen integriert [22].

Das Forschungsprojekt DIVERGesTOOL bietet Antworten auf diesen in den letzten Jahren zunehmend anerkannten Bedarf. Ziel des Forschungsprojektes war es, eine Toolbox zu entwickeln, die Forschende aus der quantitativen Gesundheitsforschung im deutschsprachigen Raum mit Fragebogen-Items und Hinweisen bei der Erfassung geschlechtlicher Vielfalt unterstützt. Im Folgenden soll zunächst das Forschungsprojekt DIVERGesTOOL vorgestellt werden. Hierauf folgt eine Erläuterung der Entwicklung der Toolbox, deren einzelne Bestandteile anschließend näher beschrieben werden. Dieser Artikel schließt mit einem Ausblick über nächste, notwendige Schritte.

## Über DIVERGesTOOL

Das Forschungsprojekt DIVERGesTOOL wurde im Zeitraum von 2020 bis 2023 durch das Bundesministerium für Gesundheit gefördert. Es wurde in interdisziplinärer Zusammenarbeit von drei verschiedenen Fachdisziplinen durchgeführt, die sich über den gesamten Studienverlauf ausgetauscht und ergänzt haben. Beteiligt waren die Disziplinen Gesundheitswissenschaften/Epidemiologie (Institut für Public Health und Pflegeforschung, Abteilung Sozialepidemiologie, der Universität Bremen), Gender Studies (Humboldt-Universität zu Berlin) und Gender Medizin (Radboud Medical Center der Universität Nijmegen). In einer Abschlussveranstaltung wurden im Sommer 2023 die Projektergebnisse der interessierten Fachöffentlichkeit vorgestellt und Implikationen für Forschung und Praxis diskutiert.

## Erstellung einer systematischen Übersicht zu existierenden Erhebungsinstrumenten

In einem ersten Schritt des Projektes wurde eine Übersicht bereits existierender Erhebungsinstrumente erstellt, die im Zeitraum von 2000 bis 2020 in der Gesundheitsforschung angewendet wurden, um verschiedene Dimensionen von Geschlecht zu erfassen [24]. Die systematische Suche in den drei Datenbanken Medline, Scopus und Web of Science ergab nach dem Entfernen von Duplikaten 5681 Treffer, für die zunächst die Titel und Abstracts und später die Volltexte von zwei voneinander unabhängigen Projektmitarbeiterinnen auf ihre Relevanz geprüft wurden. Einschlusskriterium hierbei war, dass die Instrumente in ihrem Ansatz zur Erfassung von Geschlecht über die routinemäßig eingesetzte binäre Geschlechtsvariable hinausgingen.

Insgesamt wurden 170 Studien identifiziert, innerhalb derer 77 Instrumente zur Erfassung verschiedener Dimensionen von Geschlecht angewendet wurden. Der Schwerpunkt dieser Instrumente lag auf der Erfassung sozialer Dimensionen (Gender). Gleichzeitig konnte im Zeitverlauf eine Zunahme der Vielfalt verschiedener Instrumente beobachtet werden. Insbesondere zeigte sich eine vermehrte Anwendung von Ansätzen, die soziale und biologische Dimensionen von Geschlecht miteinander kombinieren. Allerdings wurde ein großer Teil der identifizierten Instrumente in den USA oder mit einer US-amerikanischen Studienpopulation entwickelt. Hierbei handelte es sich oftmals um sehr homogene Studienpopulationen, die sich beispielsweise ausschließlich aus Studierenden zusammensetzten. Zudem hatten die meisten Instrumente ihren Ursprung im Bereich der Psychologie.

Es konnte somit ein Bedarf nach im deutschsprachigen Raum entwickelten und in deutscher Sprache formulierten Instrumenten abgeleitet werden, die in Zukunft in der gesundheitsbezogenen Forschung zur Erfassung von geschlechtlicher Vielfalt angewendet werden können. Die Ergebnisse der systematischen Übersichtsarbeit wurden in Form eines Scoping-Reviews veröffentlicht [24].

## Entwicklung eines Geschlechterkonzepts

**Abb. 1:**
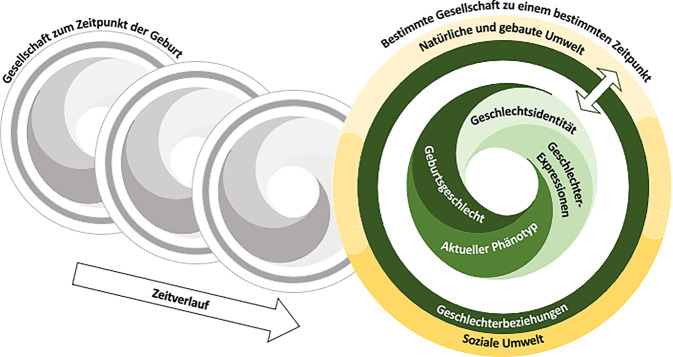
DIVERGesTOOL-Geschlechterkonzept. Ausgehend von einem multidimensionalen Verständnis von Geschlecht, setzt sich das Geschlechterkonzept aus verschiedenen sozialen und biologischen Dimensionen von Geschlecht zusammen. Diese werden dargestellt durch einzelne Ringe, die einander umschließen bzw. ineinander übergehen, um die ständige Wechselwirkung der Dimensionen zu verdeutlichen. Es lassen sich nicht immer eindeutige Grenzen zwischen den einzelnen Dimensionen ziehen, die Übergänge sind an einigen Stellen fließend. Den inneren Kern des Konzeptes bilden die eigene Geschlechtsidentität, das nach außen präsentierte Geschlecht (Geschlechterexpressionen), die derzeitigen Ausprägungen der körperlichen Eigenschaften, die mit der Reproduktion in Verbindung stehen (aktueller Phänotyp), und das Geschlecht, das bei Geburt in die Geburtsurkunde einer Person eingetragen wurde (Geburtsgeschlecht). Die Dimensionen, hier dargestellt durch spiralig angeordnete Lamellen, interagieren miteinander, ohne sich allerdings gegenseitig zu bedingen. Der innere Kern wird von einem Ring umschlossen, der die strukturelle Ebene von Geschlecht bzw. die Geschlechterbeziehungen repräsentiert. Diese beschreiben, wie soziale Beziehungen in Bezug auf das Geschlecht innerhalb einzelner Gruppen oder der gesamten Gesellschaft organisiert werden. Hierzu gehören die Rollen, Verhaltensweisen und Einstellungen, die den einzelnen Geschlechtergruppen zugeordnet werden, und die Verteilung von Macht, Privilegien und Benachteiligungen. Die individuelle und die strukturelle Ebene von Geschlecht stehen in ständiger Wechselwirkung zueinander. Weiterhin interagieren sie mit Faktoren der Umwelt, die durch den äußersten Ring der Abbildung dargestellt werden. Hierzu zählen einerseits Faktoren der sozialen Umwelt bzw. strukturelle soziale Determinanten, wie beispielsweise der sozioökonomische Status, die Bildung oder die Religion und die hiermit verbundenen Machtstrukturen. Anderseits umfasst dies Faktoren der natürlichen und gebauten Umwelt, wie die Exposition gegenüber bestimmten Umweltbelastungen. Auch die verschiedenen umweltbezogenen Einflussfaktoren sind nicht immer eindeutig voneinander abzugrenzen und gehen daher in der Darstellung fließend ineinander über. Dem Geschlechterkonzept liegt die Annahme zugrunde, dass Geschlecht fluide und abhängig von dem geografischen und zeitlichen Kontext ist, innerhalb dessen sich eine Person bewegt. Der aktuelle Zeitpunkt der Betrachtung ist in der Abbildung in Farbe abgebildet. Ergänzt wird dieser um weitere grau gefärbte, exemplarische Darstellungen vergangener Zeitpunkte im Laufe des Lebens einer Person. Dies soll aufzeigen, dass sich sowohl die sozialen als auch die biologischen Dimensionen von Geschlecht über die Zeit verändern können. Die verschiedenen Zeitpunkte überlappen einander in der Darstellung, um zu betonen, dass Expositionen aus vergangenen Lebensphasen zu einem späteren Zeitpunkt Einfluss auf die weitere Entwicklung einer Person und die sie umgebenden Strukturen nehmen können. (Quelle: DIVERGesTOOL-Projektgruppe [35])

Durch den interdisziplinären Charakter des Projektes war es zunächst von Bedeutung, ein gemeinsames Verständnis von Geschlecht als Grundlage für die Diskussion und Entwicklung von Befragungs-Items zu etablieren. Basierend auf einschlägigen Publikationen aus der internationalen Fachliteratur und der im Projektteam vertretenen Expertisen und Vorarbeiten [12] wurden zunächst Grundannahmen zu der Kategorie Geschlecht definiert, die durch das Konzept abgebildet werden sollten (Tab. 1). Diese flossen in einem nächsten Schritt in die inhaltliche Erarbeitung des Geschlechterkonzepts und dessen Visualisierung ein (Abb. 1). Das Konzept basiert auf einem multidimensionalen Verständnis von Geschlecht, das davon ausgeht, dass es verschiedene biologische und soziale Dimensionen von Geschlecht gibt, die in ständiger Wechselwirkung miteinander stehen. Die einzelnen Dimensionen sind nicht statisch, sondern können sich im Verlauf der Zeit verändern.

**Tab. 1:** Grundannahmen als Basis für das Geschlechterkonzept

Grundannahme	Erläuterung
Multidimensionalität	Es existieren mehrere verschiedene soziale und biologische Dimensionen von Geschlecht [11, 29]
Variabilität	Die einzelnen geschlechtlichen Dimensionen sind durch eine große Bandbreite von Ausprägungen gekennzeichnet [2, 11, 12]
Fluidität	Die sozialen und biologischen Dimensionen von Geschlecht sind fluide und können sich über die Zeit verändern [2]
Intersektionalität	Das Geschlecht ist nicht alleine zu betrachten, sondern steht in Wechselwirkung mit anderen sozialen Kategorien und den hiermit verbundenen Machtstrukturen und Prozessen der Privilegierung bzw. Diskriminierung [32, 33]
Embodiment	Die sozialen und biologischen Dimensionen von Geschlecht formen in Wechselwirkung den Körper, beeinflussen physiologische Prozesse und somit die Gesundheit [34]

## Aufbau eines Stakeholder-Netzwerks und partizipativer Prozess

Die Entwicklung der DIVERGesTOOL-Toolbox gestaltete sich in Form eines auf Partizipation angelegten Prozesses. Hierfür wurde ein Netzwerk aus zehn Ansprechpartner*innen und Koordinator*innen aus insgesamt acht großen epidemiologischen Studien in Deutschland etabliert. Das Netzwerk wurde zu Beginn des Projektes aufgebaut und begleitete anschließend den gesamten Entwicklungsprozess. Als potenzielle zukünftige Nutzende der Toolbox konnten die Mitglieder des Stakeholder-Netzwerks die Bedarfe und Herausforderungen ihrer individuellen Studien direkt in die Entwicklung einbringen und die Nutzer*innenfreundlichkeit und Anwendbarkeit der Items bewerten. Die Zusammenarbeit erfolgte in Form von gemeinsamen Workshops und Einzelgesprächen. Hinzu kam die Möglichkeit der schriftlichen Kommentierung. Zudem nahmen Mitglieder des Stakeholder-Netzwerks an der Abschlussveranstaltung des Projektes teil, die im Juni 2023 in Berlin stattfand.

## Entwicklung der DIVERGesTOOL-Toolbox

Aufbauend auf den Ergebnissen der systematischen Übersicht über aktuell angewendete Erhebungsinstrumente und dem im Rahmen des Projektes entwickelten Geschlechterkonzept wurde in einem nächsten Schritt eine Toolbox zur Erfassung geschlechtlicher Vielfalt für die quantitative Gesundheitsforschung erarbeitet. Aktuelle methodische Entwicklungen zur Erhebung geschlechtlicher Vielfalt wurden kontinuierlich verfolgt und flossen in die Entwicklung der Toolbox ein. Hierzu zählt beispielsweise die bereits beschriebene 2‑stufige Geschlechtsabfrage der GEDA-Studie [23].

Im Zuge von vier gemeinsam mit den Mitgliedern des Stakeholder-Netzwerks durchgeführten Online-Workshops wurden sowohl ein allgemein nutzbares Set aus Basis-Items als auch Zusatz-Items für spezifische Fragestellungen und Populationen entwickelt. Zudem wurden die entwickelten Items den Vertretungen des Bundesverbandes Trans* e. V. und dem Verein für intergeschlechtliche Menschen e. V. zur Einschätzung vorgelegt und mit diesen besprochen.

Das Content-Management-System TYPO3 CMS wurde genutzt, um die Toolbox online auf der Projekt-Website öffentlich zugänglich zu machen.

## Die DIVERGesTOOL-Toolbox

Die Fragebogeninstrumente der DIVERGesTOOL-Toolbox stehen online für alle Interessierten kostenfrei unter einer Creative-Commons-Lizenz (CC BY SA) zur Verfügung.^1^

### Basis-Items

Die Basis-Items sind ein grundlegendes, allgemein nutzbares Set aus drei verschiedenen Befragungs-Items (Infobox). Sie wurden entwickelt, um in Zukunft an der Stelle der in der Gesundheitsforschung routinemäßig zur Erfassung von Geschlecht verwendeten binären Geschlechtsvariable eingesetzt zu werden.

Das hier vorgestellte Set ist eine Weiterentwicklung einer zweistufige Geschlechtsabfrage (*Two-Step Approach*), deren Anwendung in den letzten Jahren zugenommen hat [24]. Hier werden sowohl das bei der Geburt zugeordnete Geschlecht als auch die eigene Geschlechtszugehörigkeit erfasst. Auf diese Weise können Personen, für die beide Dimensionen nicht übereinstimmen, erkannt und trans*- und cis-geschlechtliche Teilnehmende präzise identifiziert werden [19–21, 25].

Ergänzt wurden diese beiden Items für die Toolbox um eine Frage nach dem Vorliegen einer Intergeschlechtlichkeit (Diagnose „Varianten der Geschlechtsentwicklung“). Menschen mit einer Variante der Geschlechtsentwicklung weisen biologische Merkmale auf, die nicht eindeutig den bisher gängigen binären Kategorien Mann oder Frau zuzuordnen sind. Diese Varianten, die in den meisten Fällen noch immer als Krankheit klassifiziert werden, sind sehr heterogen und können sich auf sämtliche Dimensionen des biologischen Geschlechts beziehen.

Da der Geschlechtseintrag in der Geburtsurkunde meistens auf einer visuellen Begutachtung der äußeren Geschlechtsorgane bei der Geburt beruht, wird das Vorliegen einer Intergeschlechtlichkeit in vielen Fällen erst zu einem späteren Zeitpunkt im Leben erkannt [21]. Das Vorliegen einer Intergeschlechtlichkeit muss demnach explizit erfasst werden und lässt sich nicht über das Item des bei der Geburt zugeordneten Geschlechts abbilden.

Langfristig ist es denkbar, den aktuellen Geschlechtseintrag als weitere Frage zum Set der Basis-Items hinzuzufügen. Eine Änderung des Geschlechtseintrags ist in Deutschland aktuell jedoch mit rechtlichen und finanziellen Barrieren verbunden, was viele Menschen von dieser Möglichkeit ausschließt. Dies soll durch das Gesetz über die Selbstbestimmung in Bezug auf den Geschlechtseintrag (SBGG) geändert werden. Ein entsprechender Gesetzentwurf wurde vom Bundesministerium für Familie, Senioren, Frauen und Jugend und vom Bundesministerium für Justiz erarbeitet und soll im November 2024 in Kraft treten [26].

### Zusatz-Items

Die eingesetzten Instrumente zur Erfassung von geschlechtlicher Vielfalt sind abhängig von ihrem Verwendungszweck. Je nach Forschungsfrage, Studienpopulation und untersuchten gesundheitlichen Outcomes können unterschiedliche geschlechtliche Dimensionen relevant sein. Die DIVERGesTOOL-Toolbox enthält aus diesem Grund weitere, beispielhafte Fragebogen-Items für spezifische Fragestellungen oder Studienpopulationen. Die Zusatz-Items sollen Nutzenden der Toolbox als Inspiration dienen und sie dabei unterstützen, passende Items zu identifizieren, mit deren Hilfe sie die für die eigene Forschung relevanten Dimensionen von Geschlecht erfassen können.

Die Toolbox enthält Beispiele für Fragebogen-Items aus drei verschiedenen Themenbereichen bzw. für spezifische Studienpopulationen:

Zunächst wurden Items zur Abfrage durchgeführter oder geplanter Maßnahmen zur Veränderung des geschlechtsbezogenen Phänotyps entwickelt. Die Fragebogen-Items werden in zwei verschiedenen Versionen vorgestellt: In der ersten Variante werden die Gründe der Studienteilnehmenden für die Durchführung der verschiedenen Maßnahmen erfasst, während sich die zweite Version spezifisch an eine Trans*-Studienpopulation richtet. Sie kann eingesetzt werden, um mehr Informationen zu der Lebenssituation und den Motiven zu gewinnen, die mit einer Transition oder Geschlechtsangleichung einhergehen. Zudem werden mit der Toolbox drei verschiedene Items zur Erfassung von Erfahrungen mit einer falschen Geschlechtseinordnung beschrieben. Die Erfahrung, als nicht-konform mit dem eigenen Geschlecht wahrgenommen zu werden, zeigte in Studien eine Assoziation mit negativen, gesundheitsbezogenen Auswirkungen [27, 28]. Aus diesem Grund enthält die Toolbox die Abfrage, wie häufig das Geschlecht der Teilnehmenden durch andere falsch wahrgenommen wird, wie häufig das kommunizierte Geschlecht nicht respektiert wird und wie wohl sich die Teilnehmenden damit fühlen, wie ihr Geschlecht von der Außenwelt wahrgenommen wird. Darüber hinaus werden mit der Toolbox Items vorgestellt, die sich direkt an Trans*- und Inter*-Personen richten. Diese Items sollen eingesetzt werden, um die spezifische gesundheitliche Situation der Mitglieder dieser Studienpopulationen abzubilden.

### Erfassung sozialer Dimensionen von Geschlecht

Abhängig von der untersuchten Fragestellung einer epidemiologischen Studie können verschiedene soziale Dimensionen von Geschlecht relevant sein [10]. Je nachdem, ob soziale Dimensionen von Geschlecht Art und Ausmaß der zu untersuchenden Expositionen beeinflussen, die Wirkung dieser Expositionen auf die in der Studie betrachteten Gesundheitszielgrößen modifizieren oder über andere Pfade, wie beispielsweise dem Zugang zu und der Inanspruchnahme von Leistungen der Gesundheitsversorgung, mit diesen Gesundheitszielgrößen assoziiert sind, sind unterschiedliche methodische Vorgehensweisen der Datenerhebung und Datenanalyse angebracht [29]. Soziale Dimensionen von Geschlecht in epidemiologischen Studien lediglich ausschließlich als Confounder zu betrachten, wird den tatsächlichen Zusammenhängen nicht gerecht.

In der Toolbox werden die sozialen Dimensionen von Geschlecht erläutert und mögliche Zusammenhänge mit Gesundheit anhand von Beispielen illustriert. Für eine adäquate Berücksichtigung sozialer Dimensionen von Geschlecht in der Datenerhebung werden beispielhafte Leitfragen zur Verfügung gestellt, die Forschende für mögliche Geschlechtereinflüsse in ihren Forschungskontexten sensibilisieren sollen.

### Zusätzlich Hinweise und Überlegungen

Über konkrete Fragebogen-Items hinaus enthält die Toolbox weitere Hinweise und Überlegungen, die sich mit der Erfassung geschlechtlicher Vielfalt beschäftigen. Diese Aspekte werden in der Toolbox präsentiert, um Forschende bei der Auswahl geeigneter Instrumente für die von ihnen untersuchten Fragestellungen und der Durchführung einer validen, geschlechtergerechten Forschung zu unterstützen. Auch hier flossen Anmerkungen von Stakeholdern aus der Forschungspraxis und Vertreter*innen der Interessenverbände trans*- und intergeschlechtlicher Menschen ein. Thematisiert werden beispielsweise geschlechtergerechte Formulierungen im Fragebogen sowie Hinweise für die Auswahl der Studienpopulation und die Vermeidung von Diskriminierung.

## Ausblick

Die vorgestellte Toolbox zu Erfassung von geschlechtlicher Vielfalt wurde zusammen mit Vertreter*innen großer Kohortenstudien in Deutschland erstellt. Im Rahmen von vier gemeinsamen Workshops wurden erste Entwürfe der Fragebogen-Items diskutiert und weitergedacht. Bei einer Abschlussveranstaltung wurde die gesamte Toolbox der interessierten Fachöffentlichkeit vorgestellt. Die Teilnehmenden erhielten die Möglichkeit, die Items zu diskutieren und ihre Kommentare schriftlich zu hinterlassen. Alle Anmerkungen und Hinweise des Stakeholder-Netzwerks, der Interessenvertretungen Bundesverband Trans* e. V. und Intergeschlechtliche Menschen e. V. und der Veranstaltungsteilnehmenden wurden bei der Entwicklung der Toolbox berücksichtigt. Auf diese Weise ist die DIVERGesTOOL-Toolbox das Produkt aus verschiedenen Perspektiven und Expertisen. Auf Praktikabilität, d. h. Anwendbarkeit in epidemiologischen Studien hinsichtlich der benötigten Befragungszeit, wurde geachtet.

Im Rahmen des Forschungsprojektes wurden von den Stakeholdern und den Teilnehmenden der Abschlussveranstaltung häufig Bedenken bezüglich der Akzeptanz der entwickelten Fragebogen-Items thematisiert. Deutlich wurde die Befürchtung, dass große Teile der Bevölkerung die neuen Items zur Abfrage von Geschlecht nicht verstehen oder sich von diesen irritiert fühlen könnten und als Reaktion die Beantwortung dieser Items verweigern oder sogar die gesamte Befragung frühzeitig beenden könnten. Ergebnisse aus englischsprachigen Studien konnten diese Befürchtungen bisher nicht bestätigen [19, 30, 31]. So testeten Bauer und Kolleg*innen [19] die Verständlichkeit einer zweistufigen Geschlechtsabfrage, die sowohl das Geburtsgeschlecht als auch die Geschlechtsidentität der Teilnehmenden in einer kanadischen cis-geschlechtlichen Population erfasste. Die Teilnehmenden hatten keinerlei Schwierigkeiten bei der Beantwortung der Fragen und zeigten keine Ablehnung.

Aktuell gibt es noch wenig Forschung aus dem deutschsprachigen Raum, die sich mit der Akzeptanz von gendertheoretisch fundierten Geschlechts-Items beschäftigt. Erste Erfahrungen aus der bereits benannten GEDA-Studie zeigen eine hohe Akzeptanz der Two-Step-Abfrage [23]. Bislang wurde kein formaler Pretest mit der im Rahmen des Forschungsprojektes DIVERGesTOOL entwickelten Toolbox durchgeführt. Ein nächster wichtiger Schritt ist es daher, die Fragebogen-Items innerhalb verschiedener Kontexte und Populationen einzusetzen, um ihre Anwendbarkeit und Nutzer*innenfreundlichkeit umfassend zu testen.

Forschende sind dazu eingeladen, Fragebogen-Items der DIVERGesTOOL-Toolbox in ihre Forschung zu integrieren und ihre Erfahrungen zu teilen. Das Ziel ist es, auf diese Weise Hinweise aus der Anwendung der Items innerhalb verschiedener Bevölkerungen und Studienkontexte zu sammeln und auszuwerten, um die DIVERGesTOOL-Toolbox in einem iterativen Prozess stetig weiterzuentwickeln und zu ergänzen.

Die DIVERGesTOOL-Toolbox liefert einen Beitrag dazu, die Validität der Forschung zu erhöhen, indem sie Forschende dazu anregt, sich für die Anwendung progressiver Fragebogeninstrumente zur Erfassung von Geschlecht zu entscheiden. Dies ist nötig, um langfristig eine geschlechtergerechte quantitative Gesundheitsforschung zu etablieren und zu einer wissenschaftlich besser qualifizierten Gesundheitsförderung, Prävention und Gesundheitsversorgung beizutragen.

### Infobox

- **Zugeordnetes Geschlecht**
  Welches Geschlecht wurde Ihnen bei der Geburt zugeordnet (gemeint ist, welches Geschlecht bei der Geburt in Ihre Geburtsurkunde eingetragen wurde)?
  - Männlich
  - Weiblich
  - Divers
  - Es wurde kein Eintrag gemacht
- **Intergeschlechtlichkeit**
  Wurde bei Ihnen im Laufe Ihres bisherigen Lebens eine „Variante der Geschlechtsentwicklung“ ärztlich festgestellt?
  - Nein
  - Ja
- **Geschlechtszugehörigkeit**
  Bei manchen Menschen stimmt das bei der Geburt zugeordnete Geschlecht nicht mit dem Geschlecht überein, mit dem sie sich selbst identifizieren.
  Mit welchem Geschlecht identifizieren Sie sich aktuell?
  *Mehrfachantworten sind möglich*
  - Weiblich
  - Männlich
  - Trans*
  - Inter*
  - Nicht-binär
  - Agender/Kein Geschlecht
  - Genderfluid
  - Freies Textfeld/„Meine Identität wurde bei den Antwortmöglichkeiten nicht berücksichtigt“^a^

^a^Für die letzte Antwort-Kategorie des Items zur Abfrage der Geschlechtszugehörigkeit werden in der Toolbox zwei verschiedene Optionen vorgestellt: Ein freies Textfeld ermöglicht es Teilnehmenden, eine Identität außerhalb der vorgegebenen Liste zu nennen. Wird kein freies Textfeld zugelassen, kann alternativ die Antwortoption „Meine Identität wurde bei den Antwortmöglichkeiten nicht berücksichtigt“ aufgenommen werden. Durch die Auflistung wichtiger Argumente zu den Vor- und Nachteilen beider Antwortmöglichkeiten unterstützt die Toolbox Forschende bei der Auswahl einer passenden Option für die eigene Befragung.

## Footnotes

1 https://www.uni-bremen.de/divergestool-projekt/divergestool-toolbox.
